# IGF2BP3-mediated regulation of the RNA isoform and modification landscape in pancreatic cancer

**DOI:** 10.1038/s41388-026-03933-3

**Published:** 2026-08-07

**Authors:** Sandra Margielewska-Davies, Wayne Croft, Hayden Pearce, Natalie To, Fouzia Zayou, Falguni Meghrajani, Jing Zhang, Claudia M. A. Pinna, Laura Bills, Andrew D. Beggs, Soumyajit Mallick, Sarah F. Powell-Brett, Rachel Brown, Jianmin Zuo, Keith J. Roberts, Paul Moss

**Affiliations:** 1 School of Infection, Inflammation and Immunology, College of Medicine and Health, University of Birmingham, Birmingham, UK; 2 Department of Cancer and Genomic Sciences, University of Birmingham, Birmingham, UK; 3 University Hospitals Birmingham NHS Foundation Trust, Birmingham, UK; 4 Hepatobiliary and Pancreatic Surgery Unit, Queen Elizabeth Hospital Birmingham, Birmingham, UK

**Keywords:** Pancreatic cancer, Post-translational modifications, High-throughput screening

## Abstract

The incidence of pancreatic ductal adenocarcinoma (PDAC) is increasing but clinical outcomes remain poor and new treatments are required. IGF2BP3 is an oncofetal m6A-reading RNA regulatory protein whose expression is commonly observed in PDAC and which may represent an important therapeutic target. IGF2BP3 protein is expressed in >95% of primary tumours whilst RNA expression ranges from 3 to 3000-fold above normal epithelium. siRNA knockdown reveals IGF2BP3 to strongly support expression of gene programs related to DNA replication, cell cycle and apoptosis, TNF signalling and epithelial-mesenchymal transition. Direct sequencing of native RNA further reveals IGF2BP3-mediated enhancement of 863 RNA isoforms with suppression of 389 isoforms. m6A, m5C and pseudouridine (ψ) RNA modifications were seen in 97% of transcripts within the PDAC transcriptome and enriched within MYC gene targets. IGF2BP3 regulated the pattern of RNA modification with a substantial impact on m5C modifications of miRNA and scRNA, and genes associated with regulation of TNF signalling. Knockdown of IGF2BP3 expression in primary tumour organoid cultures suppressed proliferation and elicited apoptosis, indicating a critical requirement for IGF2BP3 within malignant stem cells. These data show that IGF2BP3 is expressed consistently in PDAC, plays a dominant role in regulation of RNA splicing and modification, and supports cancer stem cell proliferation and survival. IGF2BP3 therefore occupies a critical position within the RNA regulon of PDAC and represents an important therapeutic target in this tumour of unmet need.

## Introduction

Pancreatic ductal adenocarcinoma (PDAC) remains one of the greatest challenges within oncology and is predicted to become the second most common cause of cancer death within 10 years. Most patients present with locally advanced or metastatic disease and clinical outcomes remain poor. Although advances in chemotherapy, radiotherapy and surgery are being achieved, the 5-year survival following diagnosis remains <10% and new therapies are needed.

Oncofetal proteins represent important targets for cancer therapy as they are normally expressed selectively during embryogenesis, but their expression is reinduced during cell transformation. IGF2BP3 is an oncofetal protein that was originally cloned from pancreatic cancer and is a member of three related IGF2BP proteins [1]. IGF2BP3 expression is reactivated in tumours through hypomethylation [2] and positive regulation by the LINC01133 long coding RNA [3].

IGF2BP proteins are a conserved family of three RNA-binding proteins (IGF2BP1-3) with key roles in post-transcriptional gene regulation, acting to regulate mRNA stability, localisation, and translation [4–8]. Their expression is particularly high in embryonic development where they regulate cell migration, polarity and differentiation whilst also maintaining pluripotency and self-renewal. IGF2BP expression is lower in somatic tissue and this pattern is particularly notable for IGF2BP3 and underpins its potential as a selective therapeutic target in PDAC. IGF2BP3 shows selective activity for genes involved in the Ras signalling pathway [6] and inhibition of hippo signalling [9] and RNA modification plays a key role in IGF2BP3 activity. In particular, IGF2BP3 mediates its activity following binding to N6-methyladenosine (m6A)-modified RNA and is thus a functional m6A ‘reader’. m6A is the most common RNA modification across the transcriptome [10] and IGF2BP3 can thus mediate substantial impact on the RNA landscape in PDAC. IGF2BP3 has also been shown to bind to internal N7-methylguanosine (m7G) modifications where it can promote the degradation of mRNA transcripts, including *TP53* [11].

## Materials and methods

### Ethics statement

This research was approved by the Birmingham Local Research Ethics Committee REC 16/WM/0214. Fresh PDAC tissue was obtained from operative resection material with informed consent obtained from all patients. Inclusion criteria was diagnosis of pancreatic adenocarcinoma with patient undergoing potentially curative operative resection. There were no formal exclusion criteria. Sex, gender, age and weight were not determinants of recruitment and there was no study attrition. Slide sections were obtained from Birmingham Human Biomaterials Resource Centre HBRC (HTA Licence 12358), ethically approved by the North West-Haydock Research Ethics Committee; Ref 20/NW/0001, local ethics number 18–304.

### PDAC tumour vs. normal differential expression analysis on public data

Data from 7 microarrays available on NCBI (GSE15471, GSE19280, GSE28735, GSE55643, GSE62165, GSE62452 and GSE91035) were analysed to determine PDAC (vs. normal) upregulated genes using the R package limma to assess differential expression. Genes were regarded as differentially upregulated if Benjamini & Hochberg False discovery rate (FDR) < 0.05 and log two-fold change increase > 1. A Gene-set consisting of any gene found to be PDAC upregulated in at least 1 of the datasets was collated and intersected with a list of CTAg genes (265 known, 953 predicted [12]). This revealed a list of 10 CTAg genes up regulated in PDAC.

### Survival analysis

PDAC samples from TCGA were stratified into 2 groups based on high/low expression of IGF2BP3. Groupings were based on setting IGF2BP3 expression thresholds at a low (0.4) and high quintile (0.6) then selecting patients below/above these thresholds. Survival events on 70 patients in the low IGF2BP3 group were compared to 70 patients in the high IGF2BP3 group with Kaplan-Meier estimator and Cox proportional hazards (Cox PH) models using the Survival R package. CoxPH models included sex, age and stage as confounding variables in the model design formula.

### Tissue samples and paraffin sections

FFPE samples of PDAC were obtained from the University Hospitals Birmingham NHS Foundation Trust Queen Elizabeth Hospital, Birmingham, UK, via Human Biomaterial Resource Centre (HTA Licence 12358, North West-Haydock Research Ethics Committee; Ref 20/NW/0001 local ethics 18–304). Fresh pancreatic tissues were obtained with informed consent of patients under local ethics committee approval (REC no 16/WM/0214) from University Hospitals Birmingham NHS Foundation Trust, Queen Elizabeth Hospital Birmingham. Sections were cut to 4 µM in thickness and placed onto X-tra Adhesive micro slides (Surgipath Europe) by the Human Biomaterial Resource Centre.

### Cell culture, RNA extraction and cytospin preparation

All used cell lines were cultured in growth medium (CF-Pac1: IMDM + 10% FBS (Sigma-Aldrich, Gibco; Life Technologies Ltd), Panc1: DMEM + 10%FBS (Sigma-Aldrich, Gibco; Life Technologies Ltd)) at 37 °C and 5% CO_2_. Cell pellets were collected by centrifugation (1800rpm, 5 min) and RNA was extracted using Qiagen mini-RNeasy kit according to manufacturer protocol (Qiagen LTD). Cell lines were tested regularly for mycoplasma.

To prepare cytospins, a thin layer of cells was placed on a glass slide (X-tra positive charged Adhesive; Surgipath) using the Cytospin^TM^ 4 Cytocentrifuge (4 °C, at 1700 rpm for 5 min) (Thermo Fisher Scientific), and fixed with ice cold methanol for 10 min.

### Organoid culture

Pancreatic fresh tissue was enzymatically digested using enzyme cocktail of Collagenase/Hyaluronidase (1X) (StemCell Technologies LTD.), LiberaseTL (1X) and Benzonase (50U/ml) (Sigma-Aldrich) with DMEM (Sigma-Aldrich), 10% FCS (Gibco, Life Technologies Ltd.) and 1% P/S (Sigma-Aldrich). Tissue was agitated in gentleMax C tube, using GentleMACS tissue dissociator according to manufacturer protocol (Miltenyi Biotech). Cells were then seeded in the Matrigel (Corning) and cultured in growth medium (PancreaCult Organoid Growth Medium, StemCell).

### Transfection of organoids

Silencer® Select Validated siRNA from Invitrogen was used for the silencing of IGF2BP3 in cell lines and organoids (Silencer Select siRNA IGF2BP3 S20921, Negative Control No. 1 siRNA; Thermo Fisher Scientific). CF-PAC1, PANC1 and organoids cells were cultured under standard conditions (described above). Transfection was performed by nucleofection using the Cell Line Nucleofector Kit V (program R013) for cell lines, and Nucleofector kit for Primary Mammalian Epithelial Cells (program W001) for organoids (Lonza Biologicals, Slough, UK).

### qRT-PCR

#### Fluidigm®48.48 Fast Real Time PCR

The amplification of the specific target genes in cDNA samples was performed according to the manufacturer’s protocol (Fluidigm). TaqMan® Gene Expression Assays were used (Thermo Fisher). The reactions were mixed and amplified in MasterCycler® Gradient Thermal Cycler (Eppendorf). The assay was prepared according to the Fluidigm 48.48 Fast Real Time PCR Workflow Quick Reference protocol (GE 48 × 48 Standard v1 protocol) and run on BioMarkTM HD instrument for real time PCR reaction (Fluidigm). The data were collected using BioMark HD Data Collection Software v3.0.2 and analysed using Fluidigm Real-Time Software.

#### qRT-PCR reaction

All qRT-PCR reactions were prepared on 96 well optical reaction plates (Applied Biosystems, Life technologies Ltd) in a total volume of 20 µl/reaction. Each reaction was a mixture of cDNA, FastStart Universal Probe Master Mix (Roche Diagnostic Limited), 20x primer/probe of gene of interest (FAM labelled), 20x primer/probe endogenous control (GAPDH; VIC TAMRA labelled) (TaqMan^TM^, Thermo Fisher Scientific) and nuclease free water. Samples were amplified using ABI Prism 7700 Sequence Detection System (Applied Biosystems).

The threshold cycle (Ct value) data generated by both ABI Prism 7700 sequence detection system and BioMark^TM^ HD instrument were created based on amplification plots of fluorescent intensity analysed for each signal. Based on these values, the delta-delta (ΔΔ) Ct method was used, to quantify the relative levels of transcripts normalised against an endogenous control (GAPDH). The resultant value was presented as a relative gene expression, determined based on reference sample which was assigned a value of 1.

### Short read Illumina RNA sequencing

RNA from two biological replicates (24,48 and 72 h time point) of IGF2BP3 siRNA transfected pancreatic cell lines described above, was isolated, cDNA amplified, and samples sent to Genomics Birmingham facility at University of Birmingham (UK) for RNA sequencing. All samples passed quality control test and NEBNext Ultra^TM^ II Directional RNA library was produced from the amplified cDNA. In total twenty-four samples were sequenced using the NovaSeq6000 platform to give 75 base paired end read output.

### Short read Illumina RNA sequencing data analysis

Raw sequencing reads passed quality checks using FastQC and MultiQC and reads were aligned to GRCh38 human genome from gencode (GRCh38.p13.genome.fa.gz) using STAR [13] with quantMode GeneCounts. Counts matrices were imported into R (v4.2) for downstream analysis. Genes having no expression across all samples were removed. Normalisation and differential expression analysis was conducted with DESeq2 [14] to assess gene expression that was differential with respect to IGF2BP3 siRNA treatment at each time point (24, 48, 72 h). Differentially expressed genes were identified if Benjamini Hochberg false discovery rate (FDR) < 0.05. Enrichment of Hallmark gene sets from the Molecular Signatures Database was assessed using the R package fgsea for comparisons at each time point using genes ranked by log2FC. Results of gene set enrichment analysis were adjusted for multiple comparisons using the Benjamini Hochberg procedure.

### Direct RNA sequencing with oxford nanopore technologies (ONT)

Native RNA (without amplification) was extracted from 2 biological replicates for each treatment condition (control vector and IGF2BP3 siRNA) using Qiagen mini-RNeasy kit according to manufacturer protocol (Qiagen LTD). Extraction was done at the 48 h time point following transfection. Extracted RNA was sent to Genomics Birmingham facility at University of Birmingham (UK) for direct RNA library preparation and ONT sequencing using 4 PromethION flow cells (kit type: SQK-RNA004; 1 flow cell per sample). Minimum Q score was set to 9 and both FASTQ and POD5 output was generated over a 72-h run time limit.

### ONT gene and isoform expression data analysis

Raw read data from direct RNA sequencing with ONT were processed using the EPI2ME wf-transcriptomes workflow (https://github.com/epi2me-labs/) to perform genome alignment to the human genome (GRCh38 primary assembly), gene/transcript quantification and differential gene/transcript expression. Summary statistics indicating per sample mapped read number and gene/transcript counts are provided in Supplementary Table 1. Differential gene and differential isoform expression was calculated using EdgeR on gene-level and transcript-level raw counts respectively. Genes and Isoforms were considered differentially expressed between control and IGF2BP3 knockdown cells if Benjamini Hochberg FDR < 0.05.

### ONT RNA modification analysis

RNA modifications were called on each sample individually by passing pod5 files to the ONT basecaller dorado v0.8.0 (https://github.com/nanoporetech/dorado) with m6A, pseU and m5C models to generate modified bam (ModBam) files with base calls for methylated Adenine (m6A), Pseudouridine (ψ) and methylated Cytosine (m5C).

ModBam files were coordinate sorted and indexed with Samtools to prepare for further analysis with Modkit (https://github.com/nanoporetech/modkit). Summary statistic estimates of base modification proportion were calculated with *modkit summary* on a random sampling of 10,000 reads. RNA modification metrics were extracted from ModBam files and tabulated into bedMethyl files using *modkit pileup* giving a .bed file for each sample with the genomic position of all modified and unmodified bases along with read coverage metrics.

To ensure only modifications with sufficient read coverage were taken forward in downstream analysis, bedMethyl files were filtered with *awk*, keeping only base locations covered by a minimum of 6 reads.

Differential modification analysis was applied using *modkit dmr pair* to compare detected m6A ψ and m5C, modification levels in Control vs. IGF2BP3 knockdown samples. This process defines a model for the frequency of observing each base modification state and uses the binomial distribution to model the modification probability and calculates a modification likelihood ratio (score). A single base location was considered differentially modified between the sample groups if balanced MAP p-value <= 0.01.

Visualisation of modification proportions was with the R package *Gvis*. For this, filtered bedMethyl files for the 2 control and 2 knockdown samples were aggregated separately giving aggregated track data containing base location and percentage modification.

To explore genomic feature annotations specifically at modified bases aggregated bed files were filtered by >1 read coverage and percentage modified > 0% to remove unmodified loci and remove modifications with only a single read coverage. HOMER [15] *annotatePeaks with –annStats and –go* parameters was applied to annotate the filtered modification loci with genomic feature and nearest gene and to further apply hypergeometric tests calculating enrichments of genomic feature or GO term within the annotations.

### Immunofluorescence, immunohistochemical staining and Western blotting

Paraffin-embedded sections of PDAC tumour were dewaxed and dehydrated, followed by blocking of endogenous peroxidase activity in 0.3% H_2_O_2_. Citrate buffer antigen retrieval method was performed (pH 6), and slides were blocked in horse serum (Vector Laboratories). Primary antibodies were applied (IGF2BP3 (1:200, Atlas Antibodies), EPCAM (1:400, Abcam)), and slides were incubated overnight. ImmPRESS HRP reagent Universal Anti Mouse/Rabbit IgG (Vector Laboratories) secondary antibody was added. For visualisation, ImmPact DAB substrate system (Vector Laboratories Ltd) was added and slides were counterstained in Mayer’s haematoxylin (Sigma). Stained and dehydrated slides were analyzed using Nikon bright field microscope.

Immunofluorescence staining was performed using Opal 3-Plex kit (Perkin Elmer). For visualisation, Opal TSA fluorescent dyes were used (Fluorescein, Cyanine3; Perkin Elmer) according to manufacturer’s protocol. Sections were counterstained with DAPI and imaged using a Nikon E600 UV microscope (magnification 20x).

Protein expression was also analysed by standard SDS polyacrylamide gel electrophoresis and Western blot.

### Annexin V staining and viability assay

Transfected pancreatic tumour organoids seeded in Matrigel (Corning) were immersed in culture medium with Incucyte Annexin V Red Dye (1:800; Sartorius), designed to stain apoptotic cells, and incubated for the required time in 37 °C, 5%CO_2_. Annexin V expression was analysed under EVOS FL Cell Imaging system (20x magnification images; Thermo Fisher Scientific), and Incucyte S3 Live –Cell Imaging System (10x magnification images; Sartorius).

### Proliferation assay

PDAC organoids cells were transfected and seeded in Ultimatrix BME. The medium was replaced the following day, and this timepoint was defined as 0 h for subsequent analysis. To assess proliferation, cells were pulsed with 15 µM EdU for 3 h before collection at 24, 48 and 72 h. Organoids were then dissociated into single cells, stained with Zombie Red for live/dead discrimination, and fixed and permeabilized. Incorporated EdU was detected using the Click-iT Plus EdU Alexa Fluor 647 Flow Cytometry Assay according to the manufacturer’s instructions. Flow cytometry analysis was performed by gating first on GFP-positive transfected cells and then on live cells, and the percentage of EdU-positive cells within this population was used as a measure of proliferation.

### Data access

Primary data within the manuscript will be shared appropriately following requests for access. Primary analysis code was written in bash and used published tools as outlined in the Methods. Code used to generate secondary analysis results was written in R(v4.2) using published R packages as outlined in the methods. Scripts are available on reasonable request.

## Results

### IGF2BP3 is expressed in the great majority of PDAC tumours and correlates with poor prognosis

**Fig. 1:**
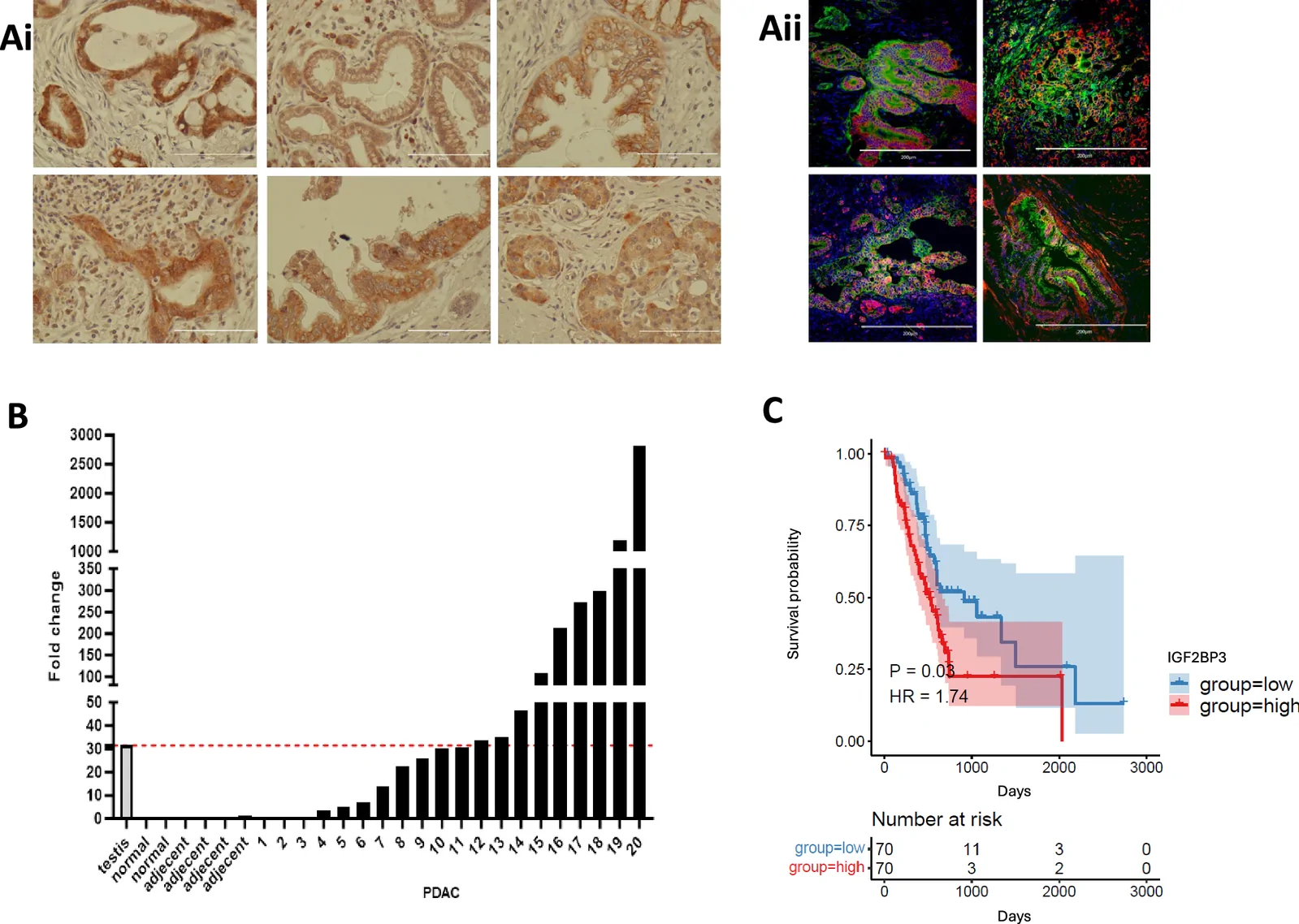
IGF2BP3 is expressed in PDAC tumour cells and expression correlates with poor clinical outcome. **A** Protein expression of IGF2BP3 in PDAC patients. (i) Representative example of immunohistochemical staining showing strong IGF2BP3 staining in 36 of 38 PDAC tumours (magnification 40x). (ii) Immunofluorescent staining on PDAC samples showing dual EPCAM (green) and IGF2BP3 (red) expression on tumour cells (double positive, yellow). Magnification 20x). **B** *IGF2BP3* gene expression in primary PDAC tumour tissue. PDAC tumours and adjacent control tissue were analysed using Fluidigm qRT-PCR (*n* = 20). Normal and adjacent pancreas tissue was added as negative control and testis as positive control (grey). **C** Overall survival is reduced in patients with high *IGF2BP3* expression. Kaplan-Meier curves of overall survival in patients with PDAC in first 3000 days from diagnosis. High expression (red) vs. low expression (blue) is compared by log-rank test. Shading represents confidence intervals.

IGF2BP3 is an important potential therapeutic target in PDAC, and initial studies assessed its expression within primary PDAC tumour tissue using immunohistochemistry. Of note, strong expression was observed in 36/38 (95%) of primary tumours. Furthermore, IGF2BP3 was localised to tumour epithelial cells with low levels of staining throughout the rest of the tumour microenvironment (Fig. 1Ai, ii). As such, IGF2BP3 is localised to tumour epithelium and represents a potential targeted therapy for PDAC.

The dynamic range of IGF2BP3 expression was next determined by assessment of relative RNA expression in a cohort of 20 primary PDAC tumours using Fluidigm qPCR (Fig. 1B). This confirmed consistent tumour-associated upregulation of *IGF2BP3* with relative expression 3- 3000-fold compared to normal tissue [16]. *IGF2BP3* expression was also associated with clinical outcome through analysis of the TCGA database (Fig. 1C) where patients were divided into high or low expression based on thresholds at the upper (60%) and lower (40%) quintiles. Cox proportional hazard test revealed that patients in the high *IGF2BP3* expression group were associated with significantly increased risk of death (HR 1.74, *p* = 0.03).

### IGF2BP3 shows variable expression within PDAC cell lines and can be suppressed by siRNA

**Fig. 2:**
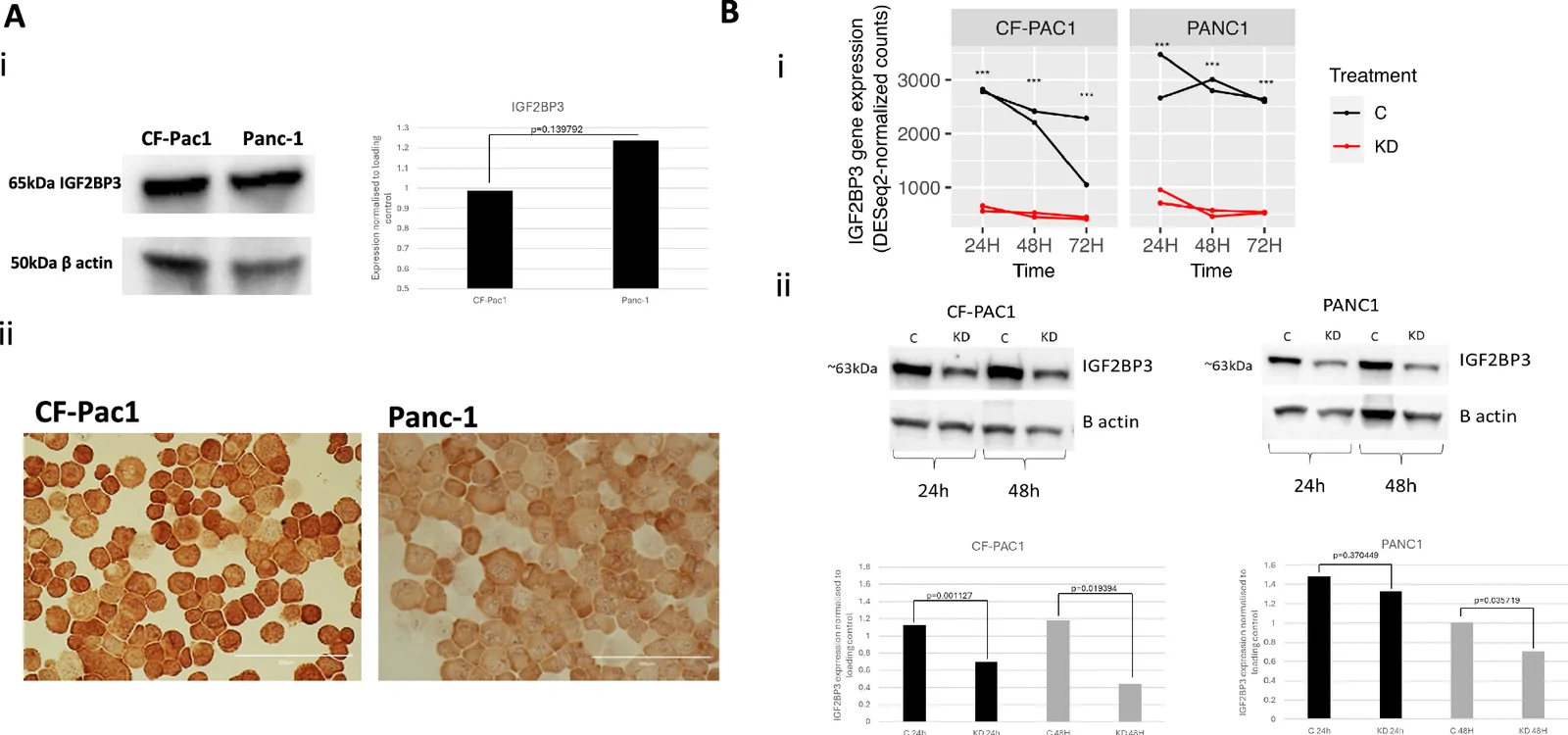
IGF2BP3 is expressed in PDAC cell lines and downregulated by siRNA. **A** (i) Western Blot and (ii) immunohistochemical analysis show strong IGF2BP3 protein expression in CF-Pac1 and PANC-1 cell lines (20x magnification). *P* value calculated based on 3 replicates. **B** Knockdown of *IGF2BP3* RNA (i) and protein (ii) in the CF-Pac1 and PANC1 cell lines. PDAC cell lines were transfected with siRNA against *IGF2BP3* and gene expression assessed by RNA sequencing at 24, 48 and 72 h in control (C) and knockdown (KD) lines (i). KD vs. C differential expression *** adjusted *p* < 0.05 *, < 0.01 **, < 0.001 ***. IGF2BP3 protein was assessed by immunoblotting at 24 and 48 h. Relative quantification was performed in ImageJ, based on 2 replicates (ii).

To facilitate mechanistic studies the level of IGF2BP3 expression was next assessed in pancreatic cancer cell lines. Low level expression was seen in Panc03.27, which was assigned a relative ratio of 1 (Supplementary Fig. 2). Compared to this, expression was increased by 21-fold in testis tissue, consistent with the profile of IGF2BP3 as a cancer-testis oncofetal protein. Increased levels of RNA expression were also seen in 6 cell lines, with a value of six-fold for PANC1 and 4200-fold increase for CF-Pac1. As such, cell lines PANC1 and CF-Pac-1 were taken forward for further analysis as representative examples of moderate and high IGF2BP3 expression (Fig. 2A). Here it was noteworthy that relative mRNA and protein expression were not strongly correlated which is likely to reflect post-transcriptional gating of translation and mRNA stability as well as relative protein stabilisation in relation to cell cycle or stress response [17].

siRNA-mediated transcriptional downregulation of *IGF2BP3* suppressed RNA levels over 72 h and was associated with reduction in IGF2BP3 protein expression (Fig. 2B). Expression of related RNA binding protein family members *IGF2BP1* and *IGF2BP3* was not altered and indicates specificity for *IGF2BP3* (Supplementary Fig. 3). Morphological analysis of cell line cultures following *IGF2BP3* knockdown did not show substantial changes in cell proliferation or survival over the subsequent two weeks (data not shown) which is not unexpected within a transformed tumour cell line.

### Downregulation of IGF2BP3 elicits widespread reduction in transcription of genes associated with proliferative activity in PDAC

**Fig. 3:**
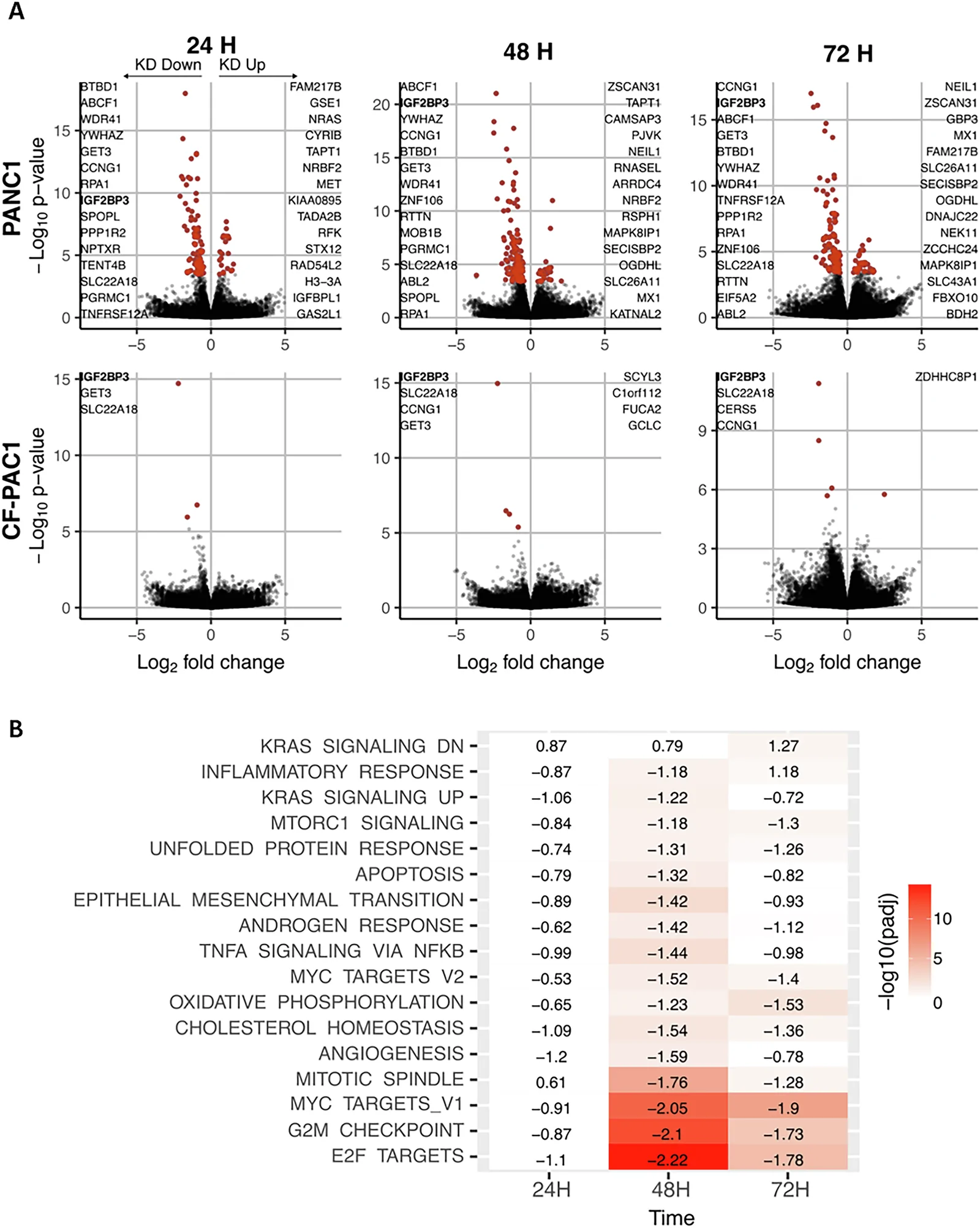
Reduced IGF2BP3 expression downregulates a wide range of genes associated with proliferation in PDAC cell lines. **A** Volcano-plot of differential gene expression following IGF2BP3 knockdown in the PANC1 and CF-PAC1 cell lines at 24, 48 and 72 h post siRNA transfection. Coloured points indicate differentially expressed genes (DEGS; adjusted *p* < 0.05) and labelled are top DEGs (ranked by *p*-value). **B** Normalised enrichment scores (NES) from Gene Set Enrichment analysis (GSEA) on transcriptome data from the PANC1 cell line indicating enrichments of selected Hallmark gene sets within up/downregulated genes at 24, 48 and 72 h post siRNA transfection. Negative NES indicates enrichments within genes downregulated following IGF2BP3 knockdown and significance is indicated by colour scale.

The impact of IGF2BP3 downregulation on the transcriptional profile of PANC1 and CF-PAC1 was next assessed using Illumina sequencing at 24, 48 and 72 h following siRNA transfection (Fig. 3A). Strong transcriptional repression of the *IGF2BP3* gene was noted and mediated widespread transcriptional downregulation (PANC1: 89, 114 and 102 genes downregulated compared to 21, 23 and 34 genes upregulated at 24, 48 and 72H respectively) In contrast, relatively few genes showed transcriptional upregulation following *IGF2BP3* knockdown. The subset of differential RNAs potentially regulated through direct binding to IGF2BP3 was assessed by intersection with RIP-seq data of IGF2BP3 targets [18]. This identified 38 binding targets within downregulated RNAs, including *IGF2BP3* itself (Supplementary Fig. 4). A single RNA, GSE1, was identified as a binding target within the upregulated genes.

Gene set enrichment analysis of Hallmark gene sets revealed that genes suppressed following *IGF2BP3* knockdown were associated with proliferation and cell division, including E2F target engagement, MYC signalling, and regulation of the G2M checkpoint and mitotic spindle (Fig. 3B). Enrichments were most prominent at 48 h post siRNA transfection and notably also included gene sets for cholesterol homeostasis, TNFA signalling, EMT and apoptosis.

**Fig. 4:**
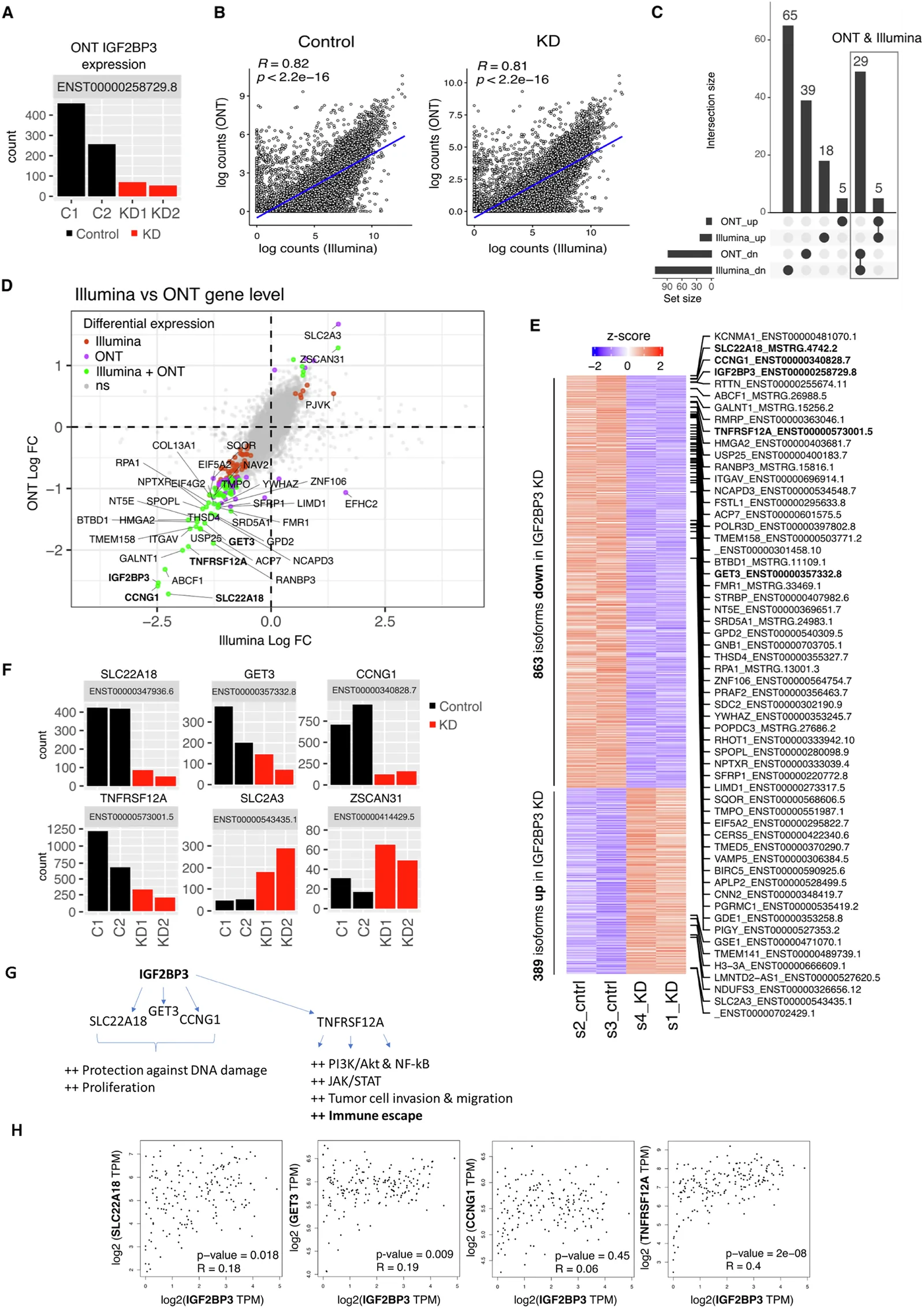
Nanopore long read sequencing identifies isoform-level IGF2BP3-regulated transcripts. **A** ONT expression counts of *IGF2BP3* transcript (ENST00000258729.8) in the PANC1 cell line at 48 h post siRNA transfection. **B** Correlation of gene level counts between Illumina cDNA and ONT direct RNA sequencing. **C** Intersection of IGF2BP3-modulated genes from Illumina and ONT sequencing. **D** *IGF2BP3* knockdown vs. Control gene level differential expression from Illumina vs. ONT transcriptome data. Point colour indicates differentially expressed genes (adjusted *p* < 0.05). **E** Differentially expressed isoforms identified from ONT transcriptome data. Labelled are the top significant isoforms (adjusted *p* < 0.0001 & absolute FC > 1). In bold are selected core consistently downregulated isoforms. **F** Normalised counts in *IGF2BP3* knockdown and control samples for selected differentially expressed isoforms **G**. Diagrammatic representation of potential core mechanisms by which IGF2BP3 may mediate tumour development by promotion of tumour proliferation and immune escape. **H** PDAC TCGA gene expression correlation of *IGF2BP3* with putative IGF2BP3-regulated genes *SLC22A18, GET3, CCNG1* and *TNFRSF12A* (*n* = 178).

The IGF2BP3-regulated transcriptome was also analysed by direct long read RNA sequencing with Oxford Nanopore Technologies (ONT) to quantify isoform expression and RNA modification landscape (Fig. 4). This enabled additional layers of information including specific isoform-level expression and RNA modification landscape (Fig. 4) that was not attainable from the short-read sequencing data. 49,392 and 47,504 transcripts were detected in control and IGF2BP3-knockdown cells respectively (Supplementary Table 1). ONT and Illumina sequencing data were confirmed to be highly correlated at the gene level (Fig. 4C, D) and direct RNA sequencing revealed knockdown of the specific *IGF2BP3* transcript ENST00000258729.8 (Fig. 4A).

Isoform-level differential expression analysis of ONT data revealed 863 and 389 isoforms down or upregulated respectively following *IGF2BP3* knockdown (Fig. 4E), further revealing IGF2BP3 as a predominantly positive regulator of RNA transcription or stability and more specifically highlighting affected gene isoforms. Relatively few isoforms were upregulated following *IGF2BP3* downregulation although this was observed for the glucose membrane transporter gene *SLC2A3* and transcription factor *ZSCAN31* (Fig. 4F).

*SLC22A18, GET3, CCNG1* and *TNFRSF12A* (Fig. 4F, G) were suppressed consistently across both cell lines detected with both Illumina and ONT technologies and thus represent a high-confidence core set of genes positively regulated by *IGF2BP3*. Transcriptome data from the TCGA database also showed high level expression of this gene set within PDAC tumours (Supplementary Fig. 5) and correlation with *IGF2BP3* expression (Fig. 4H).

### IGF2BP3 regulates the RNA modification landscape with a strong impact on miRNA and scRNA transcripts associated with p53 and TNF signalling

**Fig. 5:**
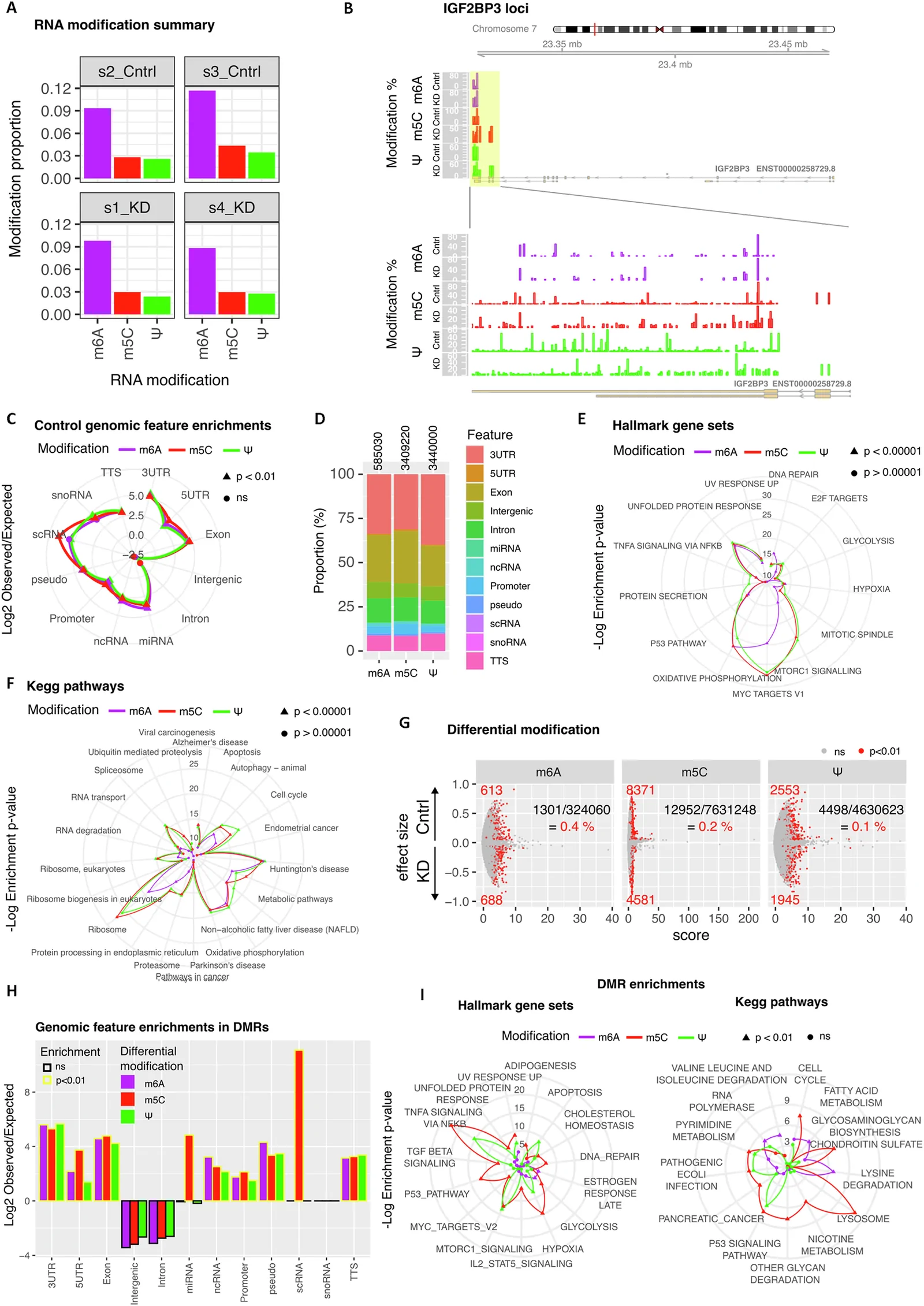
RNA modification profiles identify differential modifications following IGF2BP3 knockdown. **A** Read-level estimations of nucleoside modification frequencies within control and IGF2BP3 knockdown PANC1 cell line transcriptome from direct RNA Nanopore sequencing data. Data from randomly sampling 10k reads. **B** *IGF2BP3* genomic loci RNA modifications. Tracks show the proportions of reads with m6A, m5C and Pseudouridine (ψ) modifications from aggregated control and *IGF2BP3* knockdown sample direct RNA sequencing data. **C** Enrichment profile of genomic features within aggregated control (IGF2BP3-expressing) sample data at genomic regions identified as having m6A, m5C and ψ RNA modifications. **D** Breakdown of the proportions of genomic features found at loci with m6A (585030 sites), m5C (3409220 sites) and ψ (3440000 sites) RNA modification. Gene annotations at these RNA modification sites were assessed for enrichment of hallmark gene sets **E** and KEGG pathways **F** by hypergeometric test. **G** Differential modifications (DMRs) from control vs. *IGF2BP3* knockdown differential modification testing. Score = read coverage at the genomic position; effect size = % modified in Control - % modified in IGF2BP3 knockdown. Coloured points indicate a DMR (MAP-based *p*-value < 0.01). Arrows indicate direction of increased modification percentage in Cntrl or knockdown (KD). Summary values in red are the DMR counts in either direction and the percentage of DMRs detected at Adenine, Cytosine and Uracil sites. Enrichment of genomic features **H** Hallmark and KEGG pathway gene sets **I** within annotations at DMRs identified in **G**.

Nucleoside modifications play a critical role in RNA regulation and IGF2BP proteins are m6A-readers that enhance RNA translation and stability [19]. As such the landscape of m6A, m5C and pseudouridine (ψ) RNA modifications were next determined across the PDAC transcriptome through native RNA sequencing (Fig. 5).

Extracted RNA was directly sequenced using ONT without reverse transcription or amplification. As a result, modification marks including methylation (m6A, m5C) and pseudouridine remain present on RNA molecules that are passed through the nanopores. These perturb the ionic current signal making it subtly different from non-modified nucleoside counterparts therefore facilitating the use of pre-trained basecalling models to detect the presence of modifications from the raw signal.

m6A was the most common nucleoside modification with an estimated prevalence of ~0.09% compared to ~0.03% each for 5mC and ψ. These modifications were focussed within the 3’ UTR of *IGF2BP3* and this pattern was observed across the transcriptome, in line with known preferential localisation at RNA regulatory regions (Fig. 5B). Modifications were also focussed within miRNA and scRNAs (Fig. 5C, D).

Modification loci were next annotated for their nearest gene using genomic coordinates and assessed for gene set enrichments (Fig. 5E, F). Analysis revealed genes enriched for RNA modifications to be associated with unfolded protein response, oxidative phosphorylation, E2F and MYC targets. Variation was also observed in the distribution of the different RNA modifications across the pancreatic cancer transcriptome with m5C and ψ modifications strikingly enriched in MYC targets (Fig. 5E) and ribosomal genes (Fig. 5F).

The pattern of RNA modifications was next assessed following *IGF2BP3* knockdown to gain further insight into mechanisms through which IGF2BP3-driven RNA modification may drive oncogenesis. Given the function of IGF2BP3 as an m6A-reader protein [20] our initial assessment determined how the number and distribution of RNA modifications was modified following IGF2BP3 suppression. Over 96.5% of RNA reads contained at least one of the three modifications and this percentage did not change following IGF2BP3 knockdown. However, the impact of IGF2BP3 on the pattern of m6A, m5C or ψ modifications was observed through differential modification at 0.4%, 0.2% and 0.1% of bases respectively following IGF2BP3 knockdown (Fig. 5G). The distribution of these IGF2BP3-associated RNA modifications was next assessed across RNA regulatory regions and transcript subtypes. All three modification types were differentially regulated at regulatory regions in mRNA whilst IGF2BP3 mediated a strong impact on m5C modification pattern within microRNA (miRNA) and small cytoplasmic RNAs (scRNA) (Fig. 5H). These m5C modifications were notably enriched within transcripts associated with p53 pathway, TNF signalling and lysosome. IGF2BP3 was also seen to regulate m6A modifications in genes associated with lysine degradation (Fig. 5H, I).

These data reveal that RNA modifications are common with the PDAC transcriptome and enhanced within MYC gene targets. IGF2BP3 regulates this landscape with a notable impact on m5C-associated modification of miRNA and scRNA as well as genes associated with regulation of TNF signalling and metabolic response.

### IGF2BP3 is expressed in primary pancreatic tumour organoids and its knockdown leads to reduced proliferation and increased apoptosis

**Fig. 6:**
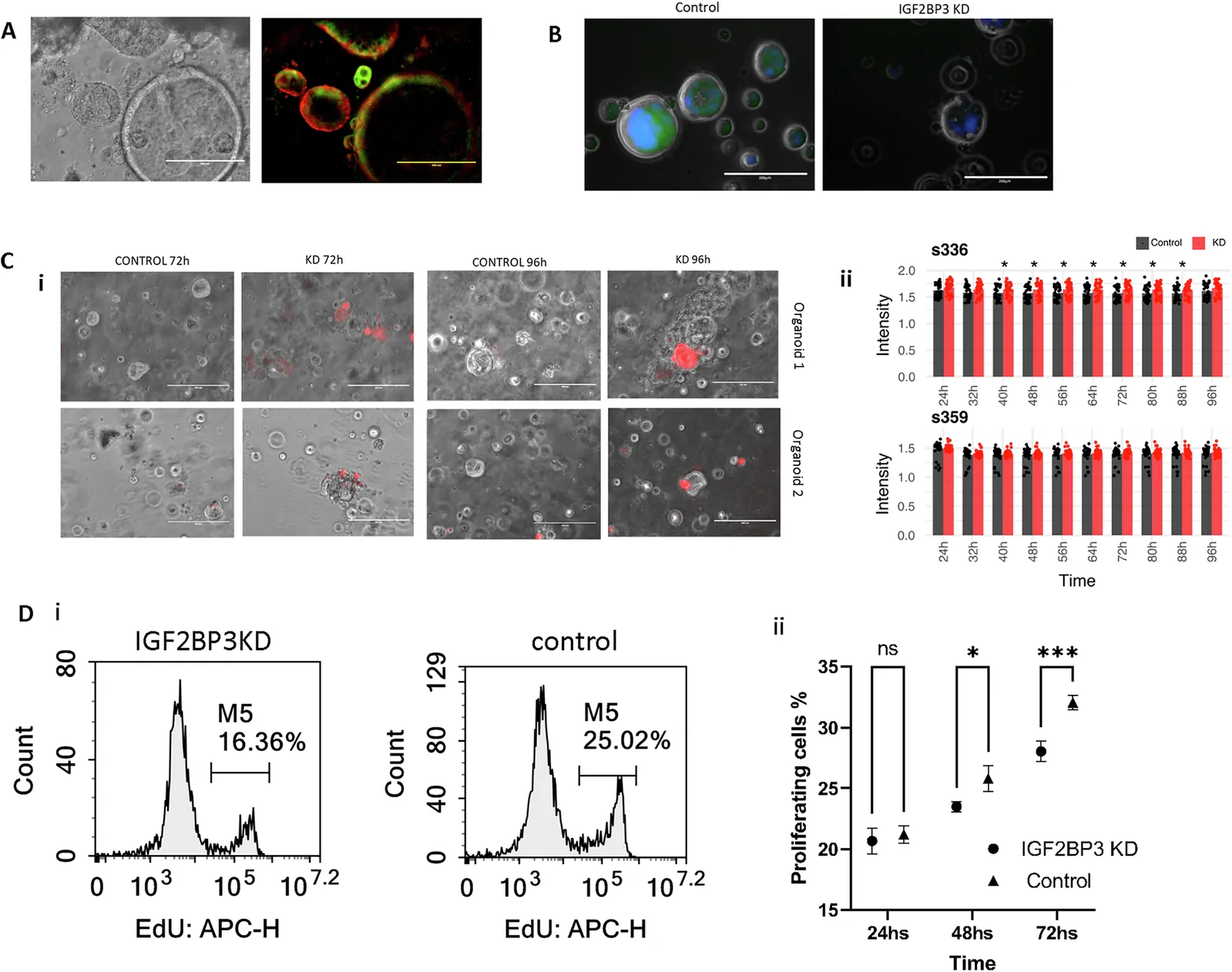
IGF2BP3 is expressed in primary pancreatic tumour organoids and its knockdown leads to reduced proliferation and increased apoptosis. **A** Expression of IGF2BP3 in PDAC organoids. Left side; brightfield image of organoid. Right side; immunofluorescence analysis with antibodies against EPCAM (red) and IGF2BP3 (green). **B** Knockdown of IGF2BP3 expression following siRNA transfection in human PDAC organoids. Immunofluorescence analysis using antibody against IGF2BP3 (green) and DAPI (blue). **C** Knockdown of IGF2BP3 expression in human PDAC organoids results in increased apoptosis as shown by (i) apoptotic bodies stained with Annexin V (red) after 72 h and 96 h from KD and (ii) 24h-96h by Incucyte S3 Live-Cell Imaging system. Graphs represent a fluorescent intensity signal for 2 independently tested biological replicates. T-test was applied within each time point to compare Control to KD distributions. **D** (i) Flow plot example of EdU staining to examine the proliferation rate of organoid cultures following *IGF2BP3* knockdown (IGF2BP3KD) or control knockdown. (ii) Proliferation rate was compared following control or *IGF2BP3* knockdown in primary PDAC organoid cultures at 24, 48 and 72 h. Data are from organoid 079 and representative of three independent experiments (Supplementary Fig. 6). Significance was analysed using two-way ANOVA and post hoc multiple comparisons, with ***< 0.001 and *p < 0.05.

IGF2BP3 has been shown to regulate a range of cellular functions in cell line 2-D model systems. Tumour organoids are a valuable resource for assessing mechanistic processes underlying malignant transformation and we next generated a series of tumour organoids from patients with primary pancreatic ductal adenocarcinoma. Immunofluorescence analysis revealed substantial IGF2BP3 expression in epithelial organoid cells (Fig. 6A). As such, the functional impact of *IGF2BP3* knockdown was assessed using electroporation of single cell organoid cultures with siRNA constructs against *IGF2BP3* and confirmed effective downregulation (Fig. 6B).

The functional impact of IGF2BP3 downregulation was next determined within organoid cultures. Annexin V immunofluorescence demonstrated substantial induction of apoptosis within organoid cultures within 72 h of siRNA knockdown. Quantitative assessment using the Incucyte S3 Live-Cell Imaging system also revealed a 40–60% increase in apoptosis within organoid cultures (Fig. 6C).

To assess proliferation, cells were pulsed with 15 µM EdU for 3 h before collection at 24, 48 and 72 h. Organoids were then dissociated into single cells, stained with Zombie Red for live/dead discrimination, and fixed and permeabilized. Incorporated EdU was detected using the Click-iT Plus EdU Alexa Fluor 647 Flow Cytometry Assay. The percentage of EdU-positive proliferative cells was then compared between the control and *IGF2BP*3 knockdown group. Proliferation was reduced in all 3 cultures at each timepoint with the great impact seen at 72 h with a relative 15–30% reduction in proliferation following *IGF2BP3* knockdown.

These data show that IGF2BP3 expression is retained in organoids where its downregulation leads to substantial reduction in proliferation and induction of apoptosis, indicating an important role in tumour stem cell survival.

## Discussion

IGF2BP3 is an oncofetal protein whose aberrant expression in somatic tissue is associated with a range of tumours. The mechanisms by which it contributes to oncogenesis are uncertain beyond its identification as an m6A-binding RNA regulatory protein. Our observations extend understanding of transcriptional regulation by IGF2BP3 and provide further support for its selection as an important therapeutic target in pancreatic cancer.

Initial studies assessed the prevalence of IGF2BP3 expression in pancreatic cancer, a key consideration in relation to its potential therapeutic utility. Here it was notable that expression was observed in 95% of tumours indicating the protein is likely an obligate component of pancreatic epithelial cell transformation [21]. Immunohistochemical analysis confirmed IGF2BP3 protein expression to be localised to tumour cells with minimal expression within the tumour microenvironment. Quantitative PCR defined the level of IGF2BP3 expression in primary tumours and showed a heterogeneous pattern with relative transcriptional levels between 3 and 3000-fold higher than in normal adjacent tissue. As such, IGF2BP3 is elevated consistently in PDAC although the determinants of this heterogeneity in relative transcription remain uncertain. Importantly, expression was correlated with clinical outcome with high IGF2BP3 expression associated with 74% increase in relative risk of death within the TCGA database [22, 23].

To assess the impact of IGF2BP3 expression on the tumour RNA transcriptome we undertook Illumina and Nanopore sequencing in the presence or absence of siRNA-mediated IGF2BP3 knockdown [18]. siRNA elicited downregulation of many genes, with enrichment of those associated with cell proliferation and division [24], and in line with the role of IGF2BP3 in enhancing RNA stability [24, 25]. MYC transcription was not altered but reduced expression was seen for MYC-regulated transcripts, indicating a novel role for IGF2BP3 in stabilising MYC target transcripts [26].

A core of three genes, SLC22A18, GET3 and CCNG1, was consistently downregulated following IGF2BP3 knockdown. SLC22A18 is a solute carrier transporter and one of only two members of this large gene family whose expression is increased in pancreatic cancer, with transcript levels above 12-fold compared to normal tissue [27]. GET3 is a chaperone protein and is not substantially implicated in PDAC although high levels correlate with bladder cancer outcome [28]. CCNG1 is a cyclin-dependent protein kinase, highly expressed in pancreatic cancer and repressed by the small non-coding RNA miR-122-5p, indicating a role for IGFBP3 in repression of ncRNA transcription [29, 30]. The membrane-associated protein TNFRSF12A, the receptor for TWEAK, was also strongly downregulated following IGF2BP3 knockdown. TNFRSF12A is overexpressed in many cancers [31] and associates with poor clinical outcome in pancreatic cancer [32], potentially identifying an underlying mechanism for the association of IGF2BP3 with immune evasion [33, 34]. siRNA knockdown was also seen to restore transcription of a range of genes that are normally suppressed by IGF2BP3 within the tumour cell. The most potent suppression was observed for SLC2A3 which is notable given that low level of expression of SLC2A3 is strongly associated with poor clinical outcome in pancreatic cancer [35]. Given that IGF2BP3 drives increased expression of SLC22A18 this reveals an important role for IGF2BP3 in regulating the profile of solute transporter expression during malignant transformation. ZKSCAN3 was also strongly suppressed by IGF2BP3 and likely relates to the importance of this transcription factor in suppressing autophagy, an important mechanism in the development of pancreatic cancer [36]. Further studies will be of value to assess the impact of sustained suppression of IGF2BP3 expression and its impact on PDAC cell biology.

Direct RNA sequencing (DRS) using Nanopore technology was also undertaken [37]. This long read sequencing allowed interrogation into IGF2BP3-related regulation of individual RNA isoforms and also delineated the pattern of RNA modification across the transcriptome [38]. Dysregulation of RNA splicing is a hallmark of cancer [39], and 863 RNA isoforms were downregulated following IGF2BP3 suppression, in line with its role as a positive regulator of transcription. However, 389 isoforms were increased after knockdown and provide insight into transcripts suppressed by IGF2BP3 during oncogenesis.

RNA nucleoside modifications play a critical regulatory role in RNA expression, transport, stability and translation and are likely of considerable importance in cell transformation [39]. DRS allows assessment of the modification pattern on RNA native strands and here we assessed the landscape of N6-methyladenosine (m6A), 5-Methylcytosine (m5C) and pseudouridine (ψ) within the pancreatic cancer transcriptome. These were present at 0.09%, 0.03% and 0.03% of bases respectively and collectively observed in 97% of RNA transcripts. Modifications were focussed within genes associated with a range of cell processes, including proliferation, and focused within RNA regulatory regions, miRNA and scRNA [40]. The distribution of individual RNA modifications across the tumour transcriptome was also variable with m5C and ψ modifications strikingly enriched in MYC target genes. Given the number of RNAs targeted by IGF2BP3 it will be important in future work to focus on the most important functional targets assessed through overexpression or silencing. The relevance of RNA modification on IGF2BP3 will also be value to assess.

The RNA modification landscape was next assessed following IGF2BP3 knockdown to determine the impact of IGF2BP3 on the pancreatic cancer transcriptome. This elicited differential modification at between 1/250 (m6A) and 1/1000 (m5C; ψ) bases. The more substantial impact at m6A sites is notable given the established role of IGF2BP3 as an m6A reader but not an m6A writer. Recent findings in leukaemia indicate that IGF2BP3 can increase m6A modifications through an increase in glycolytic flux and one-carbon metabolism, and downstream production of S-adenosyl methionine to drive methylation [20]. This mechanism may contribute to ability of IGF2BP3 to directly modulate the RNA modification landscape, which extended beyond m6A to include m5C and pseudouridine. This IGF2BP3-associated methylation footprint was focussed on mRNA regulatory regions with notable differential m5C modification of miRNA and scRNA (Fig. 5H). These IGF2BP3-related m5C modifications were enriched within genes associated with p53 and TNF signalling whilst m6A modifications were strongly associated with genes associated with lysine degradation. Importantly, this focus of RNA methylation on specific RNA transcripts supports a substrate-specific impact on RNA methylation rather than a global change. The mechanism, albeit more modest, by which IGF2P3 regulates pseudouridylation is uncertain but may include m6A-induced RNA conformational changes that increase access to PUS enzymes. Overall, these data reveal IGF2BP3 to be an important regulator of RNA modification within pancreatic epithelial cells.

IGF2BP3 was expressed broadly in primary PDAC organoid cultures, indicating its importance in sustaining pancreatic stem cell populations [12]. Here it was notable that gene knockdown suppressed proliferation and elicited substantial levels of organoid apoptosis within 72 h, further indicating the utility of therapeutic targeting, due to a focus on stem cell populations [41]. In future work it will be important to assess the impact of sustained or recurrent gene suppression on stem cell survival.

Limitations of our study include the use of cell lines for studies of IGF2BP3 knockdown. Furthermore, siRNA transduction of primary organoid cultures is challenging and did not facilitate transduction of all cells such that assessment of apoptosis likely underestimates the potential impact of IGF2BP3 suppression.

Our studies document near ubiquitous expression of IGF2BP3 in pancreatic cancer and extend insights into its role as a central transcriptional regulator of pancreatic cancer where it drives expression of a range of proliferative and cellular survival pathways. Furthermore, IGF2BP3 broadly regulates RNA isoform expression and modulates the pattern of RNA modification, linking IGF2BP3 to RNA modification type-specific pancreatic tumour cell function. IGF2BP3 is seen to be critical for proliferation and survival of primary tumour organoid cultures and together these findings further encourage therapeutic approaches to target IGF2BP3 expression in this challenging tumour subtype. Suitable approaches might include systemic delivery of siRNA or development of small molecule inhibition and in vivo validation will be critical in clinical development. Potential limitations in this regard might include compensatory transcriptional reprogramming within the tumour, potentially through the additional IGF2BP isoforms, although the consistent and high level expression of IGF2BP3 in PDAC is encouraging in this regard.

## Supplementary information

Supplementary Figures

Supplementary Table 1

## Data Availability

The datasets generated and/or analyzed in this study are available in supplementary information files or from the corresponding author on reasonable request. Illumina transcriptome sequencing data has been deposited in Gene Expression Omnibus (GSE248031) and ONT transcriptome data has been deposited in European Nucleotide Archive (PRJEB96288).
