# Supplementary material for: IGF2BP3-mediated regulation of the RNA isoform and modification landscape in pancreatic cancer
Source: Oncogene. 2026 Aug 7;45(39):4165–76. doi: 10.1038/s41388-026-03933-3 (PMC13597310; doi:10.1038/s41388-026-03933-3)
Supplement: Supplementary file 1 [file 41388_2026_3933_MOESM2_ESM.docx]

| **Sample** | **Mapped reads** | **Gene count** | **Transcript count** | **Min transcript length** | **Max transcript length** |
| --- | --- | --- | --- | --- | --- |
| C1 | 7113262 | 17646 | 51805 | 50 | 22742 |
| C2 | 5682494 | 16303 | 46979 | 34 | 22742 |
| KD1 | 7088032 | 18020 | 51091 | 34 | 19415 |
| KD2 | 6296815 | 17353 | 43917 | 34 | 18661 |

**Supplementary Table 1. Per sample summary statistics from ONT sequencing of direct RNA from the PANC1 cell line.** Summary statistics following fastq data processing with the EPI2ME wf-transcriptomes workflow. Data is from PDAC1 cell line + control vector (C; n=2) and PDAC1 cell line + IGF2BP3-targetted siRNA (KD; n=2) at 48 hours following transfection.
